# Draft Genome Sequence of Type Strain *Streptococcus gordonii* ATCC 10558

**DOI:** 10.1128/genomeA.01745-15

**Published:** 2016-02-18

**Authors:** Louise H. Rasmussen, Rimtas Dargis, Jens Jørgen Christensen, Ole Skovgaard, Xiaohui C. Nielsen

**Affiliations:** a Department of Clinical Microbiology, Slagelse Hospital, Slagelse, Denmark; b Department for Science and Environment, Roskilde University, Roskilde, Denmark

## Abstract

*Streptococcus gordonii* ATCC 10558^T^ was isolated from a patient with infective endocarditis in 1946 and announced as a type strain in 1989. Here, we report the 2,154,510-bp draft genome sequence of *S. gordonii* ATCC 10558^T^. This sequence will contribute to knowledge about the pathogenesis of infective endocarditis.

## GENOME ANNOUNCEMENT

*Streptococcus gordonii*, a member of the Mitis group streptococci, is a normal commensal in the human oral cavity (1, 2). *S. gordonii* is known to form biofilms on tooth surfaces together with other bacteria (2–5). Even though *S. gordonii* is a commensal, it can escape its niche and cause diseases, such as infective endocarditis and septic arthritis (6, 7). *S. gordonii* is known to possess genes contributing to adhesion, fibrinogen binding, and platelet binding, all of which are important factors for the pathogenesis of infective endocarditis (8–10). *S. gordonii* ATCC 10558^T^ was isolated from a patient with subacute endocarditis, and in 1989, *S. gordonii* ATCC 10558 was announced as a type strain (11). Here, we report the draft genome sequence of *S. gordonii* ATCC 10558^T^, together with the description of its genomic sequencing and annotation.

Bacterial cells of *S. gordonii* ATCC 10558^T^ were inoculated into Todd-Hewitt broth (Statens Serum Institut [SSI], Denmark) and incubated under standard conditions. Template DNA was extracted using the MasterPure Gram-positive DNA purification kit (Epicentre, USA), to which 5,000 U/ml mutanolysin from *Streptomyces globisporus* ATCC 21553 (Sigma-Aldrich, USA) was added.

Paired-end DNA libraries were constructed, with an insert size of 500 bp, and sequenced by BGI, Hong Kong, using Illumina HiSeq 2000. A total of 2,657,236 reads, with a read length of 2 × 100 bp, were assembled with SOAP*denovo* version 2.04, using a k-mer setting of 15 (12).

We obtained a draft genome of *S. gordonii* ATCC 10558^T^, composed of 66 contigs, with an *N*_50_ of 121,161 bp and an *N*_90_ of 34,144 bp. The longest contig was 323,737 bp. The estimated size of the whole genome is 2,154,510 bp (mean coverage depth, 60.53×), with a G+C content of 40.48%.

The sequence was annotated by the NCBI Prokaryotic Genome Annotation Pipeline (PGAP) (http://www.ncbi.nlm.nih.gov/genome/annotation_prok/) and by the Rapid Annotations using Subsystems Technology (RAST) server (13, 14). PGAP predicted 2,100 genes, including 1,982 coding sequences (CDSs), 31 tRNAs, 84 pseudogenes, and one 5S-16S-23S rRNA operon. In addition, PGAP predicted one clustered regularly interspaced short palindromic repeat (CRISPR). RAST allocated 52% of the genome into 338 different subsystems, of which 45 of the genes were allocated into the subsystem virulence, defense, and disease. RAST annotated CDSs for fibrinogen-binding protein, laminin-binding surface protein, and collagen adhesion protein, which all seem to be factors of importance for the pathogenesis in infective endocarditis (15–17). In addition, RAST annotated a CDS for IgA1 protease, which may cleave the human immunoglobulin A1 (18).

The GenBank entry of *S. gordonii* ATCC 10558^T^ will contribute to research on the pathogenesis of endocarditis and to the identification of Mitis group streptococci.

### Nucleotide sequence accession numbers.

This whole-genome shotgun project has been deposited in DDBJ/ENA/GenBank under the accession no. LOBS00000000. The version described in this paper is the first version, LOBS01000000.
